# The roles of *apolipoprotein E* ε4 on neuropathology and neuroinflammation in patients with Alzheimer's disease

**DOI:** 10.1111/cns.14440

**Published:** 2023-09-12

**Authors:** Mingyue He, Tenghong Lian, Peng Guo, Weijiao Zhang, Yanan Zhang, Yue Huang, Gaifen Liu, Huiying Guan, Jinghui Li, Dongmei Luo, Weijia Zhang, Wenjing Zhang, Jing Qi, Hao Yue, Xiaomin Wang, Wei Zhang

**Affiliations:** ^1^ Department of Neurology, Beijing Tiantan Hospital Capital Medical University Beijing China; ^2^ Department of Neurology, Center for Cognitive Neurology, Beijing Tiantan Hospital Capital Medical University Beijing China; ^3^ Department of Blood Transfusion, Beijing Tiantan Hospital Capital Medical University Beijing China; ^4^ Department of Pharmacology, School of Medical Sciences, Faculty of Medicine & Health UNSW Sydney Sydney New South Wales Australia; ^5^ China National Clinical Research Center for Neurological Diseases, Beijing Tiantan Hospital Capital Medical University Beijing China; ^6^ Department of Physiology Capital Medical University Beijing China; ^7^ Center of Parkinson's Disease Beijing Institute for Brain Disorders Beijing China; ^8^ Beijing Key Laboratory on Parkinson Disease Beijing China

**Keywords:** Alzheimer's disease, *apolipoprotein E* ε4, cognitive function, neuroinflammatory factors, neuropathological proteins

## Abstract

**Aims:**

To explore the roles of *apolipoprotein E* (*APOE*) ε4 on the neuropathology and neuroinflammation in Alzheimer's disease (AD) patients.

**Methods:**

AD patients were divided into the *APOE* ε4 carrier and the *APOE* ε4 non‐carrier groups according to *APOE* genotype. Demographic information, cognitive function, the levels of neuropathological proteins and neuroinflammatory factors in cerebrospinal fluid (CSF) were compared between the two groups, and their correlations were subsequently analyzed.

**Results:**

*β* amyloid protein (Aβ)_1–42_ level from the *APOE* ε4 carrier group was significantly lower than that from the non‐carrier group (*p* = 0.023), which was associated with worse cognitive function. The nitric oxide (NO) level was significantly elevated in the *APOE* ε4 carrier group compared to the non‐carrier group (*p* = 0.016), which was significantly and positively correlated with the Trail Making Test (TMT)‐A‐time (*r* = 0.21, *p* = 0.026) and TMT‐B‐time (*r* = 0.38, *p* < 0.01).

**Conclusion:**

*APOE* ε4 is associated with poorer cognition, particularly the early symptoms of memory, language, and attention. *APOE* ε4 is associated with lower Aβ_1–42_ level, and the more numbers of *APOE* ε4 are carried, the lower level of Aβ_1–42_ is measured. *APOE* ε4 is associated with elevated NO level, which is linked to the impaired attention and executive function.

## Graphical abstract

*Apolipoprotein E (APOE)* ε4 is associated with poorer early symptoms of Alzheimer's disease (AD). The more numbers of *APOE* ε4 are carried, the lower level of β amyloid protein (Aβ)_1–42_ is measured. *APOE* ε4 is associated with elevated nitric oxide (NO) level, which is linked to impaired attention and executive function.

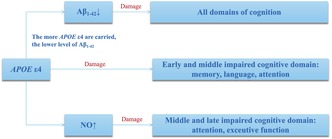

## 1 INTRODUCTION

Alzheimer's disease (AD) is the most common neurodegenerative disease in the elderly, which is characterized by progressive cognitive decline, neuropsychiatric symptoms, and impairments of daily activities.

The pathological hallmark of AD includes neuritic plaques and neurofibrillary tangles, which are composed of *β* amyloid protein (Aβ) and phosphorylated tau (P‐tau), respectively. Aβ and P‐tau, as the biomarkers of AD neuropathology, are very pivotal in the development and progression of AD.^1^

Neuroinflammation also plays an important role in AD.^2^, ^3^ Microglia and astrocytes make up the majority of glial cells, which transform into different subtypes after being stimulated and then play either a pro‐inflammatory or anti‐inflammatory role.^4^ This is manifested as overactivation of glial cells, which turn into a pro‐inflammatory phenotype, and then produce numerous neuroinflammatory factors, including tumor necrosis factor (TNF)‐α, interleukin (IL)‐1β, IL‐6, interferon and nitric oxide (NO), which promote the development of AD pathology and induce progressive degeneration and death of neurons.^2^

The human *Apolipoprotein E (APOE)* encodes the essential lipid transporter APOE in the brain. There are three types of alleles of *APOE*, including ε2, ε3, and ε4, and *APOE* ε4 is the highest risk gene for sporadic AD.^5^ Compared with other *APOE* alleles, *APOE* ε4 carriers showed a higher risk of developing sporadic AD, earlier onset age, higher rate of cognitive decline, and poorer cognitive function.^5^, ^6^, ^7^

The roles of *APOE* ε4 on the neuropathology of AD were investigated. It was reported that *APOE* ε4 is more potent than other *APOE* alleles in inducing amyloid precursor protein (APP) transcription, promoting the transformation of Aβ peptide into neurotoxic Aβ oligomer and fibrils, and inhibiting the clearance of Aβ in the brain.^8^, ^9^ Moreover, *APOE* ε4 carriers had more tau aggregation and a greater extent of somatodendritic tau redistribution in the brain in comparison to other *APOE* alleles.^10^ In addition, several animal model studies explored that the extent of glial hyperplasia in the brain and levels of TNF‐α, IL‐1β, and IL‐6 of *APOE* ε4 carriers were greater than that of other *APOE* alleles.^10^, ^11^

Nevertheless, there are still many problems remaining with *APOE* ε4 and AD. Evidence suggests that *APOE* ε4 is associated with poorer cognitive function than other alleles, however, the association between *APOE* ε4 and various cognitive domains involved remains unclear. Furthermore, studies related to *APOE* alleles with pathological mechanisms of AD have predominantly focused on animal models, autopsy studies, or PET studies. It is well established that extrapolating results from animal model studies to clinical patients can occasionally be challenging, and that intracerebral pathological changes shown from autopsy and PET frequently occur later than those detected by CSF. Consequently, the discovery of a link between *APOE* allele and factor changes in the CSF of AD patients at an earlier stage is currently understudied and particularly crucial. In addition, some recently identified neuroinflammatory factors, including chitinase‐3‐like protein 1 (CHI3L1, also known as YKL‐40) and triggering receptor expressed on myeloid cells 2 (TREM2), were shown alteration in AD.^12^, ^13^ However, it is still unclear how these factors are related to *APOE* alleles in AD.

This study hypothesized that *APOE* ε4 might promote the processes of AD by accelerating the aggregation of neuropathological proteins and upregulating levels of neuroinflammatory factors. Based on this hypothesis, AD patients enrolled in the study were divided into the *APOE* ε4 carrier and non‐carrier groups. Demographic information, cognitive function, the levels of neuropathological proteins of AD and neuroinflammatory factors in CSF between the two groups was compared, and the correlations among the above‐mentioned variables were subsequently analyzed.

## 2 MATERIALS AND METHODS

### 2.1 Subjects

Patients who were diagnosed with AD according to the National Institute of Aging and Alzheimer's Association (NIA‐AA) criteria were consecutively enrolled in this cross‐sectional study from the Center for Cognitive Neurology, Department of Neurology, Beijing Tiantan Hospital, Capital Medical University.^14^, ^15^ The exclusion criteria were as follows: (1) Patients with neurological diseases that might affect cognition besides AD, including frontotemporal dementia, dementia with Lewy bodies, corticobasal degeneration, Parkinson's disease, multiple sclerosis, and epilepsy, etc. (2) Patients with severe systematic diseases, including heart failure, pulmonary diseases, and kidney failure, etc. (3) Patients with active systemic infections, including pulmonary infection and urinary tract infection, etc. (4) Patients with chronic infectious diseases, malignancy, autoimmune disease or are being treated with steroids, etc. (5) Patients suffered traumatic brain injury recently. (6) Patients undergone major surgery.

### 2.2 Collection of demographic information

Demographic information was collected, including gender, age, age of onset, education level, drinking, smoking, body mass index (BMI), resting heart rate, blood pressure, history of hypertension, history of hyperlipidemia and history of diabetes mellitus, etc.

### 2.3 Assessments of cognitive function

Global cognitive function of patients was assessed by the Mini‐Mental State Examination (MMSE) and the Montreal Cognitive Assessment (MoCA) scales. In terms of functions of individual cognitive domains, verbal memory was evaluated by the Auditory Verbal Learning Test (AVLT) and visual delayed memory was evaluated by the Rey‐Osterreithm Complex Figure Test (RFT)‐delayed recall.^16^, ^17^ Language was evaluated by the Verbal Fluency Test (VFT) and the Boston Naming Test (BNT).^18^, ^19^ Attention was evaluated by the Symbol Digit Modalities Test (SDMT),^20^ the Trail Making Test (TMT)‐A,^21^ as well as the Stroop Color‐Word Test (SCWT)‐A and SCWT‐B.^22^ Visuospatial ability was evaluated by RFT. Executive function was evaluated by the SCWT‐C and the TMT‐B. The detailed description of these scales was provided in the Appendix S1.

### 2.4 Detection of APOE genotypes

The venous blood samples of enrolled patients were collected from the median elbow under fasting condition the next morning after admission, and then sent to the clinical laboratory of Beijing Tiantan Hospital.

Genotyping for *APOE* single nucleotide variants (rs429358 C/T and rs7412C/T), which define the *APOE* 𝜀2, 𝜀3, and 𝜀4, was performed by real‐time Fluorescence Quantitative Polymerase Chain Reaction by using nucleic acid detection reagents (Youzhiyou company).

### 2.5 Collections of CSF samples

All patients in our study were first‐time visitors and had not used cognitive‐improving drugs such as cholinesterase inhibitors before admission, thus excluding the influence of drugs on the results. Lumbar puncture was conducted and CSF samples were collected under fasting condition through lumbar puncture, followed by being immediately centrifuged at 4°C with 3000 r/min for 10 min. Each CSF sample was then allocated into separate Nunc cryotubes (Beijing JingkeHongda Biotechnology Co., Ltd) and frozen for 0.5 mL per tube at −80°C until the assay.^23^

### 2.6 Measurements of neuropathological proteins of AD in CSF

Neuropathological proteins of AD, including Aβ_1–42_ (CSB‐E10684h kit, CUSABIO Company), total tau (T‐tau) (CSBE12011h kit, CUSABIO Company), P‐tau (T181) (KHB7031 kit, Invitrogen), P‐tau (S199) (KHO0631 kit, Invitrogen), P‐tau (T231) (KHB7041 kit, Invitrogen), and P‐tau (S396) (KhB8051 kit, Invitrogen) were detected by ELISA.

### 2.7 Measurements of neuroinflammatory factors in CSF

Neuroinflammatory factors, including TNF‐α (Human TNF alpha Uncoated ELISA kit, Invitrogen), IL‐1β (Human IL‐1 beta Uncoated ELISA kit, Invitrogen), IL‐6 (Human IL‐6 Uncoated ELISA kit, Invitrogen), IFN‐γ (Human IFN gamma Uncoated ELISA kit, Invitrogen), sTREM2 (Human TREM2 DuoSet ELISA, R&D Systems), and YKL‐40 (ProcartaPlex™ Immunoassay Kit, Invitrogen) were detected by ELISA. NO (A013‐2‐1 kit, Nanjing Jiancheng Biological Engineering Research Institute) and hydroxy radical (OH) (A018‐1 kit, Nanjing Jiancheng Biological Engineering Research Institute) were measured by chemical colorimetry.^24^

### 2.8 Statistical analysis

Statistical analyses were performed by SPSS Statistics 21.0 (IBM Corporation). Statistical significance was defined as a two‐sided *p* < 0.05.

Data were tested for normal distribution using the Kolmogorov–Smirnov test. Demographic variables, cognitive function, the levels of neuropathological proteins of AD and neuroinflammatory factors between the *APOE* ε4 carrier and non‐carrier groups were compared. Continuous variables conforming to normal distribution were presented as means ± standard deviations (SD) and compared by two‐tailed *t* test, while non‐normal distributed measurement variables were presented as median (quartile) and compared by nonparametric test, and categorical variables were presented as number (percentage) and compared by Chi‐Squared test. The correlation of cognitive function with neuropathological proteins of AD and neuroinflammatory factors in CSF were performed by Spearman's correlation analysis and presented by heat maps. Multiple linear regression analysis were further performed to adjust for confounding variables.

## 3 RESULTS

### 3.1 The frequency of the *APOE* ε4 in AD patients

**FIGURE 1:**
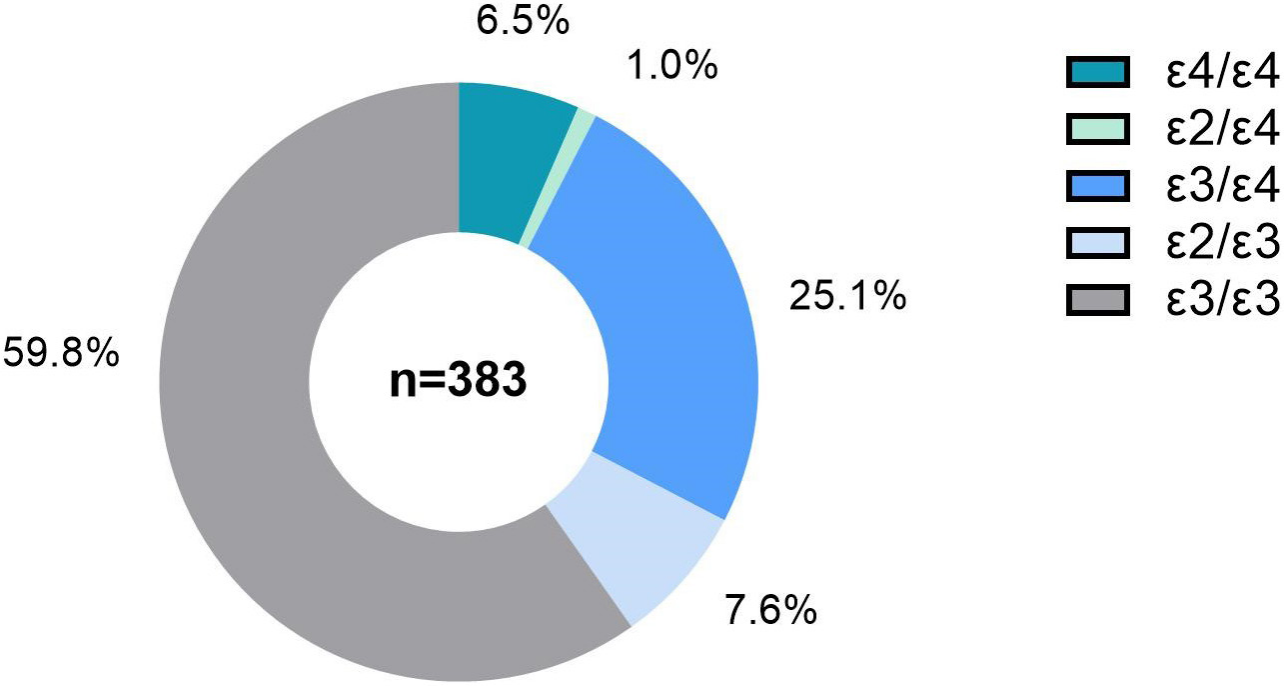
The frequency of *apolipoprotein E* (*APOE*) genotypes in patients with Alzheimer's disease (AD). There are five *APOE* genotypes in this cohort, including ε2/ε3 (7.6%), ε2/ε4 (1.0%), ε4/ε4 (6.5%), ε3/ε4 (25.1%), and ε3/ε3 (59.8%).

A total of 383 AD patients were enrolled in this study, 125 cases (32.6%) carried *APOE* ε4, among whom four cases (1.0%) carried the *APOE* ε2/ε4, 96 cases (25.1%) carried the *APOE* ε3/ε4, and 25 cases (6.5%) carried the *APOE* ε4/ε4. In addition, 29 cases (7.6%) carried the *APOE* ε2/ε3, and the *APOE* ε3/ε3 was the most frequent one (59.8%) (Figure 1).

### 3.2 Association of *APOE* ε4 with clinical features

In this study, 125 cases (32.6%) were the *APOE* ε4 carrier group, in which 76 cases (60.8%) were female and the median age was 68.00 (62.00, 74.00) years. The data showed no significant differences in demographic variables between the two groups (Table 1). The median disease duration was 24.00 (12.00, 48.00) months in the *APOE* ε4 carrier group, which was not significantly different from the non‐carrier group. The *APOE* ε4 carrier group had a significantly higher frequency of AD‐D than the *APOE* ε4 non‐carrier group (*p <* 0.001). (Table 1).

**TABLE 1:** Clinical features of the *APOE* ε4− and *APOE* ε4+ groups.

	*APOE* ε4− group (*n* = 258)	*APOE* ε4+ group (*n* = 125)	*p*
Female [*n* (%)]	149 (57.75)	76 (60.80)	0.617
Age [years, median (quartile)]	66.00 (60.00, 74.00)	68.00 (62.00, 74.00)	0.150
Age of onset [years, median (quartile)]	62.00 (55.00, 70.00)	65.00 (58.00, 72.00)	0.143
Education level [*n* (%)]			0.109
Primary school and below	54 (20.93)	27 (21.60)	
Middle and high school	126 (48.84)	60 (48.00)	
Bachelor's degree and above	78 (30.23)	38 (30.40)	
Smoking [*n* (%)]	61 (23.64)	26 (21.00)	0.519
Drinking [*n* (%)]	53 (20.54)	24 (19.20)	0.792
BMI [median (quartile)]	23.89 (21.89, 26.11)	23.40 (21.30, 24.90)	0.087
Resting heart rate [Times/minute, median (quartile)]	72.00 (69.50, 78.00)	72.00 (69.00, 76.00)	0.344
Systolic blood pressure [mmHg, median (quartile)]	132.00 (122.00, 143.00)	133.50 (124.75, 148.50)	0.195
Diastolic blood pressure [mmHg, median (quartile)]	81.00 (75.00, 87.75)	83.00 (75.75, 90.00)	0.336
History			
Hypertension [*n* (%)]	102 (39.53)	43 (34.40)	0.404
Hyperlipidemia [*n* (%)]	49 (18.99)	29 (23.20)	0.489
Myocardial infarction [*n* (%)]	3 (1.16)	1 (0.80)	0.892
Atrial fibrillation [*n* (%)]	2 (0.78)	2 (2.00)	0.512
Diabetes mellitus [*n* (%)]	49 (18.99)	17 (13.60)	0.253
Hyperhomocysteinemia [*n* (%)]	3 (1.16)	16 (12.80)	0.897
Cerebrovascular disease [*n* (%)]	37 (14.34)	10 (10.00)	0.119
Sleep apnea syndrome [*n* (%)]	14 (5.42)	6 (4.80)	0.954
Depression [*n* (%)]	16 (6.20)	11 (8.80)	0.686
Disease duration [years, median (quartile)]	24.00 (12.00, 48.00)	24.00 (12.00, 48.00)	0.730
Stage of disease [*n* (%)]			
MCI	150 (58.14)	50 (40.00)	0.001**
Dementia	108 (41.86)	75 (60.00)	
Cognitive function			
Global cognitive function			
MMSE [points, median (quartile)]	22.00 (15.00, 26.00)	19.00 (11.00, 24.50)	0.005**
MoCA (points, mean ± SD)	15.01 ± 7.44	12.93 ± 7.41	0.011*
Individual cognitive domain			
Memory			
AVLT N1‐3 (points, mean ± SD)	11.46 ± 6.04	8.98 ± 5.67	<0.001**
AVLT N4 [points, median (quartile)]	1.00 (0.00, 4.00)	0.00 (0.00, 2.00)	<0.001**
AVLT N5 [points, median (quartile)]	0.00 (0.00, 4.00)	0.00 (0.00, 2.00)	<0.001**
RFT delayed recall [points, median (quartile)]	3.00 (0.00, 10.00)	0.00 (0.00, 8.00)	0.027*
Language			
VFT (points, mean ± SD)	33.05 ± 16.64	26.88 ± 16.06	<0.001**
BNT [points, median (quartile)]	23.00 (18.00, 26.00)	21.00 (14.00, 25.00)	0.023*
Attention			
SDMT (points, mean ± SD)	21.09 ± 15.27	19.83 ± 21.41	0.571
TMT‐A time (seconds, mean ± SD)	105.99 ± 72.95	128.59 ± 76.28	0.012*
SCWT‐A time [seconds, median (quartile)]	40.00 (30.00, 54.00)	49.89 (27.00, 60.00)	0.925
SCWT‐B time [seconds, median (quartile)]	53.00 (39.00, 72.50)	55.50 (40.43, 80.00)	0.620
Visuospatial ability			
RFT imitation [points, median (quartile)]	27.25 (8.75, 34.00)	22.00 (2.00, 33.00)	0.255
Executive function			
SCWT‐C time (seconds, mean ± SD)	107.97 ± 73.25	102.81 ± 61.18	0.554
TMT‐B‐time [seconds, median (quartile)]	207.00 (122.00, 240.00)	240.00 (161.00, 240.00)	0.050

*p < 0.05, **p < 0.01.

Abbreviations: *APOE* ε4−, *APOE* ε4 non‐carriers; *APOE* ε4+, *APOE* ε4 carriers; *APOE*, *apolipoprotein E*; AVLT, Auditory Verbal Learning Test; BMI, body mass index; BNT, Boston Naming Test; MCI, mild cognitive impairment; MMSE, Mini‐Mental State Examination; MoCA, Montreal Cognitive Assessment; RFT, Rey‐Osterrieth Complex Figure Test; SCWT, The Stroop Color and Word Test; SDMT, Symbol Digit Modalities Test; TMT, Trail Making Test; VFT, Verbal Fluency Test.

Cognitive function was also compared between the two groups. As far as global cognitive function, the scores of MMSE (*p* = 0.005) and MOCA scales (*p* = 0.011) were all significantly decreased in the *APOE* ε4 carrier group compared with that in the non‐carrier group. Besides, each individual cognitive domain between the two groups was also conducted. Firstly, in terms of the memory, the scores of AVLT N1‐3 (*p <* 0.001), AVLT N4 (*p <* 0.001), AVLT N5 (*p <* 0.001), and RFT‐delayed recall (*p* = 0.027) in the *APOE* ε4 carrier group were all significantly lower than those in the non‐carrier group. Secondly, in terms of the language, the *APOE* ε4 carrier group had significantly decreased scores of VFT (*p <* 0.001) and BNT scales (*p* = 0.023) compared with the non‐carrier group. As far as the attention, the *APOE* ε4 carrier group spent more time on the TMT‐A test (*p* = 0.012) and the TMT‐B test (*p* = 0.050) than the non‐carrier group did. There was no significant difference in executive function and visuospatial ability between the two groups.

### 3.3 Association among *APOE* ε4, neuropathological proteins in CSF, and cognition in AD patients

#### 3.3.1 Neuropathological proteins between the *APOE* ε4 carrier and the non‐carrier groups

Aβ_1–42_ level in CSF from the *APOE* ε4 carrier group was significantly lower than that from the non‐carrier group (*p* = 0.023) (Table 2). There were no statistical differences in the levels of P‐tau (T181), P‐tau (S199), P‐tau (T231), P‐tau (S396) and T‐tau in CSF from the *APOE* ε4 carrier group than that from the non‐carrier group. Multiple linear regression analyses showed *APOE* ε4 was negatively associated with Aβ_1–42_ level after adjusting for age, gender, and disease duration [*β*, −1.13; 95% CI (−2.15, −0.11); *p* = 0.031].

**TABLE 2:** Levels of neuropathological proteins between the *APOE* ε4− and *APOE* ε4+ groups.

	*APOE* ε4− group	*APOE* ε4+ group	*p*
Aβ_1–42_ [ng/mL, median (quartile)]	0.79 (0.44, 1.09)	0.63 (0.35, 1.00)	0.023*
P‐tau (T181) [ng/mL, median (quartile)]	58.25 (31.94, 89.84)	72.30 (37.94, 90.47)	0.737
P‐tau (S199) [ng/mL, median (quartile)]	6.43 (4.29, 11.34)	7.21 (4.11, 11.77)	0.932
P‐tau (T231) [ng/mL, median (quartile)]	81.79 (64.74, 107.60)	86.10 (59.45, 112.16)	0.239
P‐tau (S396) (ng/mL, mean ± SD)	66.38 ± 27.47	60.21 ± 28.17	0.949
T‐tau [ng/mL, median (quartile)]	89.12 (72.62, 122.93)	94.83 (66.18, 123.83)	0.365

*p < 0.05.

Abbreviations: *APOE* ε4−, *APOE* ε4 non‐carriers; *APOE* ε4+, *APOE* ε4 carriers; *APOE*, *apolipoprotein E*; Aβ, *β* amyloid protein; P‐tau, phosphorylated tau; T‐tau, total tau.

**FIGURE 2:**
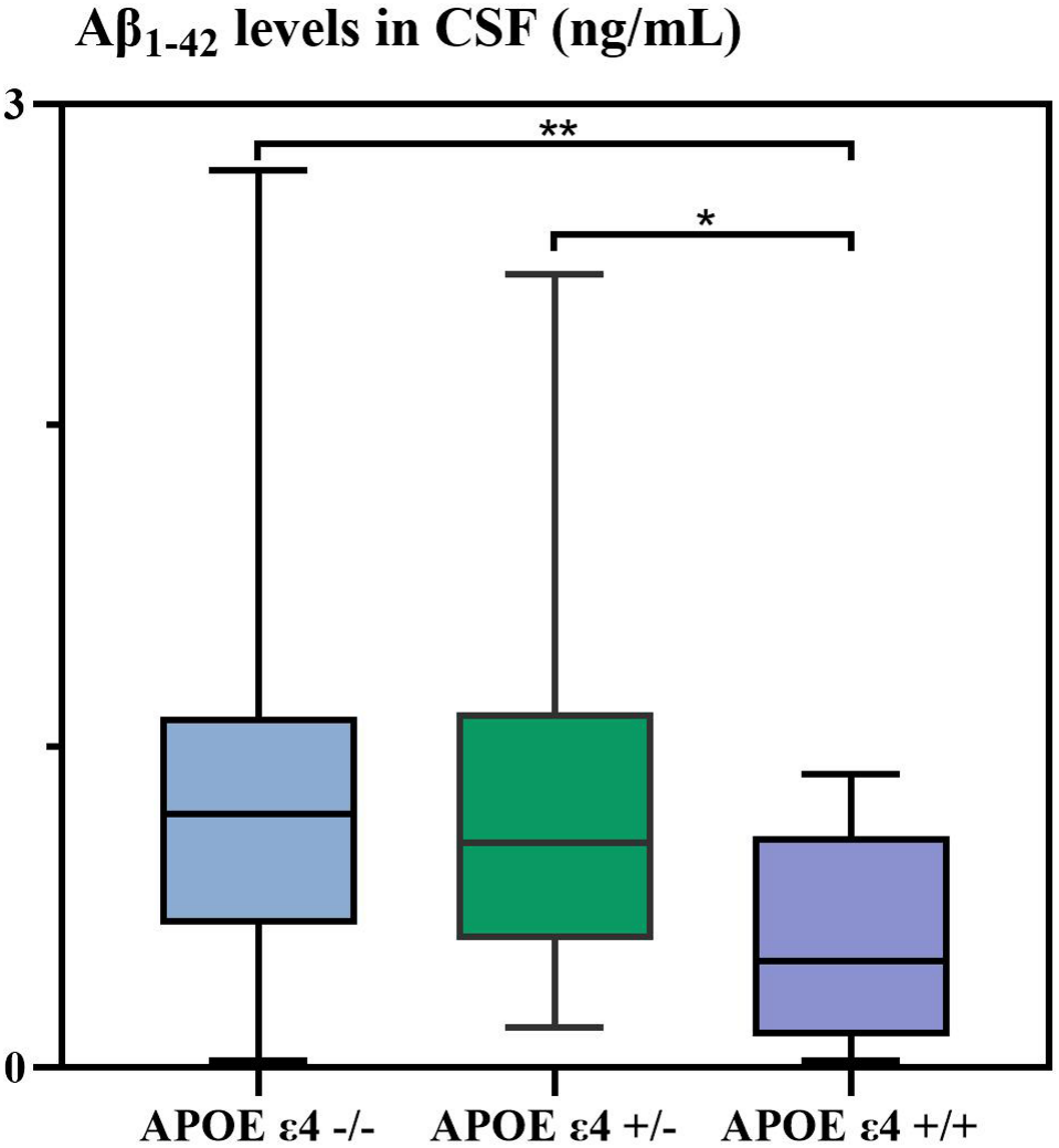
Aβ_1–42_ level between the groups of *APOE* ε4−/−, *APOE* ε4+/− and *APOE* ε4+/+. *APOE* ε4+/−, single *APOE*ε4 allele carriers; *APOE* ε4+/+, double *APOE* ε4 alleles carriers; *APOE*, *apolipoprotein E*; Aβ, *β* amyloid protein; *APOE* ε4−/−, *APOE* ε4 allele non‐carrier. *APOE* ε4−/− versus *APOE* ε4+/+, *p < 0.05; *APOE* ε4+/− versus *APOE* ε4+/+, **p < 0.01.

Aβ_1–42_ level from the *APOE* ε4−/−, the *APOE* ε4+/−, and *APOE* ε4+/+ groups were compared (Figure 2). In comparison to the *APOE* ε4−/− group, Aβ_1–42_ level in CSF was reduced in the *APOE* ε4+/− group (*p* = 0.879) and significantly decreased in the *APOE*ε4+/+ group (*p* = 0.011). In addition, compared with the *APOE* ε4+/− group, Aβ_1–42_ level was markedly declined in the *APOE*ε4+/+ group (*p* = 0.009).

### 3.4 Correlations between Aβ_1‐42_ level and cognition

**FIGURE 3:**
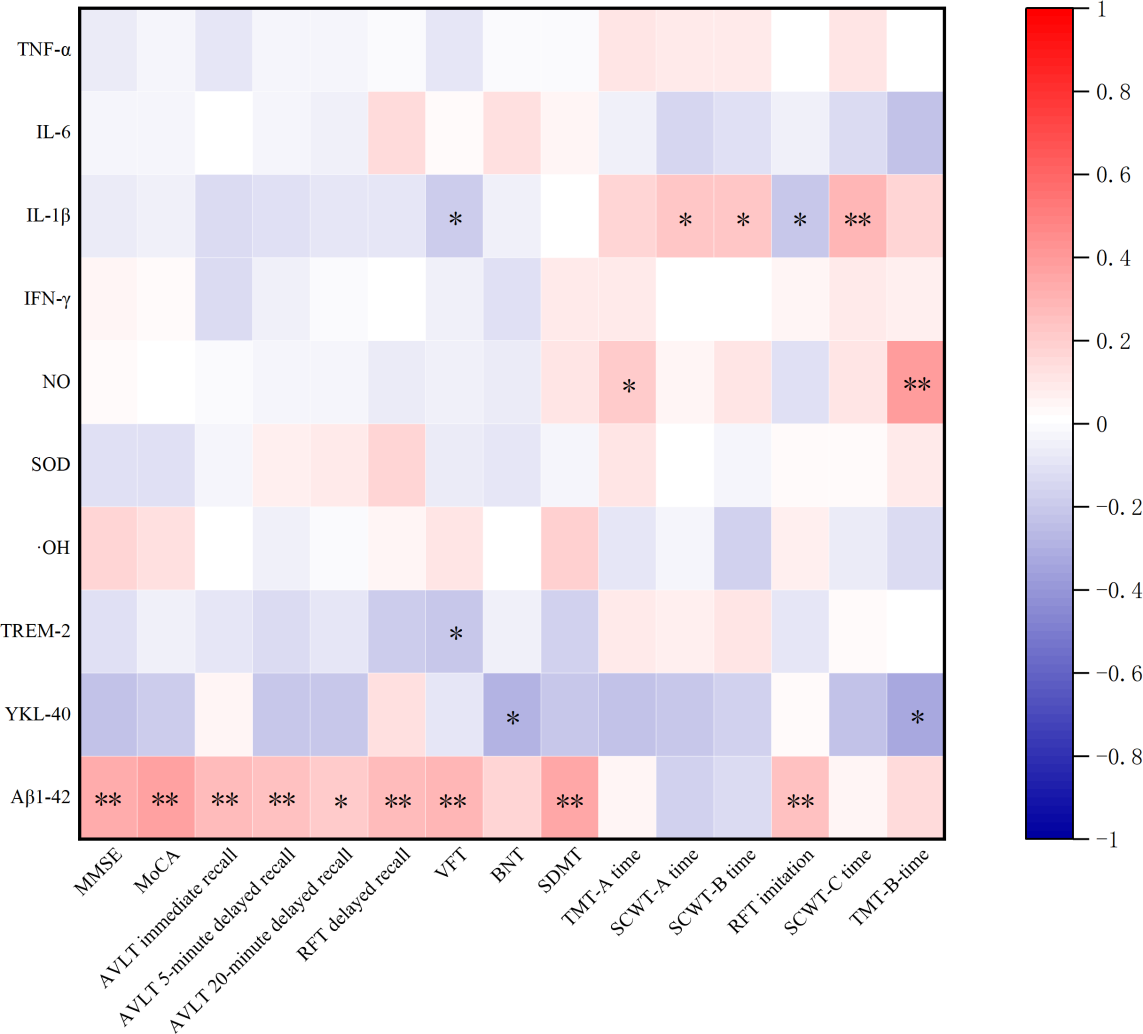
Heat map of correlations between neuroinflammatory factors, Aβ_1–42_ and cognition in AD patients. AVLT, Auditory Verbal Learning Test; Aβ, *β* amyloid protein; BNT, Boston Naming Test; IFN‐γ, interferon‐γ; IL‐1β, interleukin‐1β; IL‐6, interleukin‐6; MMSE, Mini‐Mental State Examination; MoCA, Montreal Cognitive Assessment; NO, nitric oxide; OH, hydroxy radical; RFT, Rey‐Osterrieth Complex Figure Test; SDMT, Symbol Digit Modalities Test; SCWT, The Stroop Color and Word Test; sTREM‐2, triggering receptor expressed on myeloid cells‐2; TMT, Trail Making Test; TNF‐α, tumor necrosis factor‐α; VFT, Verbal Fluency Test; YKL‐40, Tyr‐Lys‐Leu‐40. *p < 0.05, **p < 0.01.

In the aspect of overall cognitive function, Aβ_1–42_ level in CSF was significantly and positively correlated with the scores of MMSE (*r* = 0.32, *p <* 0.001) and MoCA (*r* = 0.36, *p <* 0.001) scales (Figure 3).

In terms of individual cognitive domains, Aβ_1–42_ level was also significantly and positively correlated with the scores of AVLT N1‐3 (*r* = 0.26, *p* = 0.003), AVLT N4 (*r* = 0.25, *p* = 0.006), AVLT N5 (*r* = 0.20, *p* = 0.026), RFT‐delayed recall (*r* = 0.27, *p* = 0.005), VFT (*r* = 0.30, *p <* 0.001), SDWT (*r* = 0.34, *p <* 0.001), and RFT imitation (*r* = 0.25, *p* = 0.009). These results suggested that more Aβ_1–42_ deposition in the brain was significantly correlated with the dramatically declined overall cognitive function and multiple cognitive domains of memory, language, attention, and visuospatial ability in *APOE* ε4 carriers. There were no marked correlations between Aβ_1–42_ level and executive function (Figure 3).

Multiple linear regression analyses further illustrated that lower Aβ_1–42_ level was associated with worse overall cognitive function and individual cognitive domains, including memory, language, and attention, which was independent of age, disease duration, and education level (Table S1).

### 3.5 Association among *APOE* ε4, neuroinflammatory factors in CSF, and cognitive function

#### 3.5.1 Association between *APOE* ε4 and the levels of neuroinflammatory factors

Multiple linear regression analyses were conducted to explore associations between *APOE* ε4 and levels of neuroinflammatory factors, including TNF‐α, IL‐1β, IL‐6, IFN‐γ, NO, OH, sTREM2, and YKL‐40 in CSF. It was found that *APOE* ε4 was markedly associated with elevated NO level in CSF after adjusting for age, gender and disease duration [*β*, 2.24; 95% CI (0.18, 4.30); *p* = 0.033] (Table 3).

**TABLE 3:** Association between levels of neuroinflammatory factors and *APOE* ε4 in AD patients.

	Unadjusted		Adjusted	
	*β* (95%CI)	*p*	*β* (95%CI)	*p*
TNF‐α (pg/mL)	2.08 (−0.17,4.33)	0.070	2.14 (−0.22, 4.50)	0.075
IL‐1β (pg/mL)	0.24 (−0.69, 1.16)	0.618	0.16 (−0.81, 1.14)	0.744
IL‐6 (pg/mL)	0.02 (−0.43, 0.48)	0.918	−0.02 (−0.49, 0.44)	0.920
IFN‐γ (pg/mL)	−0.11 (−1.99,1.77)	0.906	−0.27 (−2.26, 1.72)	0.787
NO (μmol/L)	2.24 (0.18, 4.30)	0.033*	2.58 (0.40, 4.77)	0.021*
OH (U/mL)	−42.85 (−101.50, 5.81)	0.151	−52.03 (−113.04, 8.98)	0.094
STREM‐2 (pg/mL)	−72.30 (−240.23, 95.69)	0.395	−82.51 (−246.54, 81.51)	0.321
YKL‐40 (pg/mL)	5764.47 (−6400.52, 17929.44)	0.347	3564.20 (−9368.21, 16496.62)	0.583

Age, gender, disease duration, and education level were adjusted. AD, Alzheimer's disease; *APOE* ε4‐, *APOE* ε4 non‐carriers; *APOE* ε4+, *APOE* ε4 carriers; *APOE*, *apolipoprotein E*; IFN‐γ, interferon‐γ; IL‐1β, interleukin‐1β; IL‐6, interleukin‐6; NO, nitric oxide; OH, hydroxy radical; sTREM‐2, triggering receptor expressed on myeloid cells‐2; TNF‐α, tumor necrosis factor‐α; YKL‐40, Tyr‐Lys‐Leu‐40. *p < 0.05.

### 3.6 Correlations between neuroinflammatory factors and cognition

Correlations between the levels of neuroinflammatory factors in CSF and the scores of cognitive rating scales in AD patients were displayed (Figure 3). The NO level was significantly and positively correlated with TMT‐A‐time (*r* = 0.21, *p* = 0.026) and TMT‐B‐time (*r* = 0.38, *p <* 0.01), demonstrating that higher NO level was significantly correlated with worse attention and executive function. Moreover, IL‐1β level in CSF was significantly and negatively correlated with the scores of VFT (*r* = −0.18, *p* = 0.043) and RFT‐imitation scales (*r* = −0.21, *p* = 0.025), while it was significantly and positively correlated with the time spent on SCWT‐A (*r* = 0.22, *p* = 0.017), SCWT‐B (*r* = 0.23, *p* = 0.013), and SCWT‐C (*r* = −0.29, *p <* 0.01) in AD patients, indicating that the elevated IL‐1β level was markedly correlated with impaired language, visuospatial ability, attention, and executive function. Additionally, the levels of sTREM2 and YKL‐40 were all significantly and negatively correlated with worse language function (*p <* 0.05).

Associations between neuroinflammatory factors in CSF and cognitive function of AD were further validated by linear regression analysis and adjusted for possible confounding factors, including age, disease duration, and education level **(**Tables S2‐S5). The results showed that the higher NO level was also associated with longer time of TMT‐A [*β*, 3.24; 95% CI (0.00, 6.47); *p* = 0.050] and TMT‐B [*β*, 5.24; 95% CI (1.11, 9.37); *p* = 0.013] after adjusting for the above confounding factors (Table S2). Furthermore, the higher IL‐1β level was associated with longer time of SCWT‐A [*β*, 8.10; 95% CI (2.51, 13.68); *p* = 0.005] and SCWT‐C [*β*, 6.61; 95% CI (0.27, 12.94); *p* = 0.041], which were independent of age, disease duration, and education level (Table S3). In addition, YKL‐40 level was significantly and negatively associated with the score of BNT [*β*, −1.30E‐4; 95% CI (−2.26E‐4, 3.30E‐5); *p* = 0.011], which was independent of age, disease duration, and education level (Table S5). The predictive value of sTREM2 for VFT was not found (Table S4).

## 4 DISCUSSION

### 4.1 Frequency of the *APOE* ε4 in AD patients

In this study, 32.6% of total AD patients carried *APOE* ε4, among whom, 6.5% carried double *APOE* ε4. The frequency of *APOE* ε4 in this study was higher than that in Hispanic (24.0%) and Asian (28.0%) populations reported by previous studies,^25^, ^26^ which might be due to the different ethnic and national backgrounds of the enrolled subjects.

### 4.2 *APOE* ε4 aggravated cognitive impairment of AD patients

In this study, no difference in demographic variables, including age of onset were found between the two groups, which differed from previous study showing that *APOE* ε4 carriers had an earlier onset age of AD compared with other *APOE* alleles carriers.^27^ The differences might be related to the different race, age, and duration of disease of the population between our and other investigations. Large‐scale epidemiological study from China is required to clarify the link between *APOE* ε4 and demographic variables, such as age, age of onset, and gender, etc.

In this study, *APOE* ε4 was obviously associated with poorer cognitive function. AD patients with *APOE* ε4 had considerably worse overall cognitive function than those without *APOE* ε4. Previous studies demonstrated that AD patients with *APOE* ε4 had poorer cognitive function, particularly the significantly severe memory impairment,^28^, ^29^ which was possibly because *APOE* ε4 was associated with temporal lobe atrophy (especially hippocampus) and dysfunction in the default mode network.^29^, ^30^ Furthermore, we applied multiple cognitive tests to comprehensively evaluate individual domains and found that AD patients carrying *APOE* ε4 were markedly impaired in language and attention in addition to memory. It is well known that memory, language, and attention are the cognitive domains involved in the early stage of AD, suggesting that *APOE* ε4 might play a key role in the early stage of AD.

### 4.3 *APOE* ε4 aggravated cognitive impairment via neuropathological proteins of AD

In this study, Aβ_1–42_ level in CSF was significantly and positively correlated with both overall cognitive function and multiple cognitive domains, including memory, language, and attention, suggesting that the higher Aβ burden in the brains of AD patients, the worse the cognitive function.

In a previous animal model study, *APOE* ε4‐targeted replacement familial AD (EF4AD) transgenic mice had more Aβ deposition in brain than E3FAD mice had.^31^ A longitudinal study based on Aβ‐PET imaging showed that AD patients with *APOE* ε4 had diffusely increased accumulation of Aβ pathology through the cortex.^32^ The clearance of soluble Aβ in brain depended on the transport of low‐density lipoprotein receptor‐related protein 1 (LRP1), hence, the binding of LRP1 to APOE protein blocked the clearance of soluble Aβ. APOE4 protein encoded by *APOE* ε4 had the highest binding ability to LRP1, and thus had the strongest blocking effect on Aβ clearance.^9^ In addition, *APOE ε4* had a greater effect than other *APOE* alleles on enhancing APP transcription, promoting transformation of Aβ peptide into neurotoxic Aβ oligomer and fibrils, prolongating half‐life of Aβ and inhibiting enzymatic degradation of Aβ in the brain of AD animal models^33^, ^34^ and AD patients.^8^ These data further demonstrated that *APOE* ε4 played an important role on AD pathology in the early stage of disease. Nevertheless, clinical studies on the relationship between *APOE* ε4 and Aβ in CSF were insufficient, although the alterations of neuropathological protein in CSF generally precede imaging changes.

Hence, we particularly focused on the association between *APOE* ε4 and Aβ_1–42_ level in CSF in AD patients, and found that *APOE* ε4 was markedly and negatively correlated with the significantly decreased Aβ_1–42_ level in CSF, and innovatively demonstrated that quantitative dependence of *APOE* ε4 on Aβ_1–42_ level in CSF. That is, the more numbers of *APOE* ε4 that AD patients carried, the heavier brain burdens of Aβ_1–42_ that AD patients had.

This study observed no obvious correlation between *APOE* ε4 and tau pathology in AD patients. Previous studies revealed that *APOE* ε4 carriers had greater tau aggregation in the brain of AD animal models^35^, ^36^ and AD patients^37^ compared to other *APOE* allele carriers.^35^, ^36^, ^37^ Nevertheless, there is still a lack of studies on the relationship between *APOE* alleles and tau pathology in CSF. The subjects included in this study were mainly in the early stage of AD (the median disease duration was 24 months), which might be the reason that the correlation between *APOE* ε4 and tau level in CSF was not found.

### 4.4 Neuroinflammation exacerbated cognitive impairment of AD patients

In this study, the increased levels of multiple neuroinflammatory factors in CSF were associated with the compromised cognitive function of AD patients. In detail, the enhanced NO level in CSF was associated with worsened attention and executive function, the elevated IL‐1β level in CSF was linked to the impaired language, attention, visuospatial ability, and executive functions, and the increased YKL‐40 level was related to compromised language function. The above data suggested that neuroinflammation exacerbated the functions of overall cognition and multiple cognitive domains of AD patients.

Neuroinflammation featured by the overactivation of glial cells, robustly produced a body of neuroinflammatory factors, including TNF‐α, IL‐6, IL‐1β and NO, etc., and subsequently led to neuronal damage and cognitive impairment.^38^ We found that the elevations of neuroinflammatory factors exacerbated the cognitive impairment of AD patients, and different neuroinflammatory factors were involved in different cognitive domains, indicating that these neuroinflammatory factors might play distinct roles in different stages of AD.

Recently, a variety of novel neuroinflammatory factors, such as astroglia‐expressed YKL‐40 and microglia‐expressed TREM2, have been discovered. It was found that the increased YKL‐40 level in CSF predicted the faster cognitive decline in the early stage of AD.^12^, ^39^ In this study, high level of YKL‐40 in CSF was associated with compromised language function, one of the early impaired cognitive domains in AD, suggesting that overactivation of astroglia might occur in the early stage and was one of the upstream mechanisms of AD. In addition, results from AD mouse models suggested that TREM2 deficiency increased the volume of neuritic plaques in brain, induced tau hyperphosphorylation, promoted neuroinflammation, and exacerbated cognitive impairment.^40^, ^41^, ^42^ Nevertheless, the correlation between sTRME2 level in CSF and cognitive function of AD patients was not understood, and the roles of sTREM2 in the different stages of AD patients remain unclear. Research derived from AD mouse models demonstrated that TREM2 might play a deleterious role in the earlier stage and a protective role in the later stage of AD.^43^ In this study, we found that higher sTREM2 level in CSF was associated with poorer language function. The median disease duration of patients included in this study was 24 months, indicating that sTREM2 might exert a negative effect on cognitive function in the early stage of AD.

### 4.5 *APOE* ε4 aggravated cognitive impairment of AD patients via neuroinflammation

The current study showed that *APOE* ε4 independently predicted the higher NO level in CSF from AD patients. As one of the critical redox active species and a free radical participating in both oxidative and nitrosative reactions, the NO level is increased because of inflammatory activation and oxidative stress in AD.^44^ Previous studies on AD mouse models discovered that *APOE* ε4 significantly drove microglia to transform into the classic activated type (M1 type) and release magnitude of proinflammatory factors, including NO, compared with other *APOE* alleles.^44^, ^45^ However, whether *APOE* ε4 can affect NO level in CSF of AD patients and the precise mechanism by which *APOE* ε4 promotes NO production are not yet known. Our study was innovative in finding an association between *APOE* ε4 and NO level in CSF from AD patients, whereas the underlying mechanisms need to be further explored.

In this study, *APOE* ε4 was not significantly associated with levels of YKL‐40 and sTREM2 in CSF from AD patients. A previous study found the relationships between *APOE* ε4 and YKL‐40,^46^ the mechanisms underlying their relationships were still unclear. We speculate that the differences in the results among individual investigations may be due to the different race and disease duration of the enrolled patients. Although the positive correlation between APOE and TREM2 was found in animal models of AD,^47^ investigations on the relationships between *APOE* ε4 and sTREM2 in CSF from AD patients are still lacking. Further studies are needed to explore the association of *APOE* ε4 with YKL‐40 and sTREM2 in CSF from AD patients.

### 4.6 Limitations

This study has the following limitations. Measurement of variables in CSF is one of the most objective ways reflecting the pathophysiological changes in the brains of AD patients. However, multiple factors make it challenging to acquire CSF from the elderly, particularly from people in normal cognitive condition, and we will increase CSF samples from AD patients and collect CSF samples from cognitively normal controls in the future. In addition, this is a cross‐sectional study, thus, further longitudinal studies with large samples will be performed to explore the dynamic influence and underlying mechanism of *APOE* ε4 on neuropathology and neuroinflammation in AD patients.

## 5 CONCLUSION

Totally 32.6% AD patients carry *APOE* ε4. *APOE* ε4 is associated with poorer cognitive function of AD, particularly the early symptoms of memory, language, and attention. *APOE* ε4 is associated with lower Aβ_1–42_ level in CSF, and the more numbers of *APOE* ε4 are carried, the lower level of Aβ_1–42_ is measured. *APOE* ε4 is associated with the elevated NO level in CSF, which is linked to the impaired cognitive domains of attention and executive function. Results from this investigation help understand the mechanism underlying the pathogenesis of AD and cast a new light in terms of the pivotal roles of *APOE* ε4 on neuropathology and neuroinflammation in patients with AD.

## AUTHOR CONTRIBUTIONS

Mingyue He contributed to the conception, design, and data statistics of the study and paper writing; Tenghong Lian, Peng Guo, Weijiao Zhang, Huiying Guan, Jinghui Li, Dongmei Luo, Weijia Zhang, Wenjing Zhang, Jing Qi, and Hao Yue contributed to the acquisition and collation of data; Yue Huang, Yanan Zhang, Gaifen Liu, and Xiaomin Wang contributed to direct paper writing and data statistics; Wei Zhang contributed to the conception and design of the study and the supervision of paper writing.

## FUNDING INFORMATION

This research was supported by The National Key R&D Program of China‐European Commission Horizon 2020 (2017YFE0118800‐779,238); The National Key Research and Development Program of China (2016YFC1306300, 2016YFC1306000); The National Natural Science Foundation of China (81,970,992, 81,571,229, 81,071,015, 30,770,745, 82,201,639); Capital's Funds for Health Improvement and Research (CFH) (2022–2‐2048); The Key Technology R&D Program of Beijing Municipal Education Commission (kz201610025030); The Key Project of Natural Science Foundation of Beijing, China (4161004); The Natural Science Foundation of Beijing, China (7082032); Project of Scientific and Technological Development of Traditional Chinese Medicine in Beijing (JJ2018‐48); Capital Clinical Characteristic Application Research (Z121107001012161); High Level Technical Personnel Training Project of Beijing Health System, China (2009‐3‐26); Project of Beijing Institute for Brain Disorders (BIBD‐PXM2013_014226_07_000084); Excellent Personnel Training Project of Beijing, China (20071D0300400076); Project of Construction of Innovative Teams and Teacher Career Development for Universities and Colleges Under Beijing Municipality (IDHT20140514); Beijing Healthcare Research Project, China (JING‐15‐2); Basic‐Clinical Research Cooperation Funding of Capital Medical University, China (2015‐JL‐PT‐X04, 10‐JL‐49, 14‐JL‐15); Natural Science Foundation of Capital Medical University, Beijing, China (PYZ2018077); Youth Research Funding, Beijing Tiantan Hospital, Capital Medical University, China (2015‐YQN‐14, 2015‐YQN‐15, 2015‐YQN‐17).

## CONFLICT OF INTEREST STATEMENT

The authors report no competing interests.

## Supporting information


Appendix S1

## Data Availability

The data that support the findings of this study are available from the first author or the corresponding author upon reasonable request.
