# Supplementary material for: The roles of apolipoprotein E ε4 on neuropathology and neuroinflammation in patients with Alzheimer's disease
Source: CNS Neurosci Ther. 2023 Sep 12;30(3):e14440. doi: 10.1111/cns.14440 (PMC10916449; doi:10.1111/cns.14440)
Supplement: Supplementary file 1 — Appendix S1 [file CNS-30-e14440-s001.docx]

Supplementary material

# Assessments of cognitive function

## Global cognitive function

Global cognitive function of AD patients was assessed by the Mini-Mental State Examination (MMSE) and the Montreal Cognitive Assessment (MoCA) scales. Patients with illiteracy, primary education, or more than a junior education were identified as dementia when the MMSE score was below 17, 20 or 24 points, respectively. The MoCA score ≤ 26 indicated potential cognitive impairment and 1 point was added if the educational level of an individual was less than 12 years. The lower the scores of the two scales, the worse the global cognitive impairment.

## Individual cognitive domain

### Memory

Verbal memory was evaluated by the Auditory Verbal Learning Test (AVLT), which consists of 5 learning trials in total^1^. During each trial, a list of 12 novel words was read aloud with a 1-second pause between each word, and the subjects were asked to recall immediately as many words as possible. The above process was repeated 3 times, with scores named N1, N2, and N3, respectively. The subjects were then asked to recall the 12 words again after a 5-minute and 20-minute interval, with scores named N4 and N5, respectively. Immediate recall, short-delayed recall and long-delayed recall were evaluated as AVLT N1-3, AVLT N4 and AVLT N5. Poor verbal delayed memory was indicated by the low score of AVLT.

Visual delayed memory was evaluated by the Rey-Osterreithm Complex Figure Test (RFT)-delayed recall^2^. In the test, the subject were asked to duplicate a complex figure within 10 minutes, with scores named RFT-imitation, and then drew the figure from memory after 25 minutes, with scores named RFT-delayed recall^2^. Poor visual delayed memory was indicated by the low score of RFT-delayed recall.

### Language

Language function was evaluated by the Verbal Fluency Test (VFT) and the Boston Naming Test (BNT) . VFT consists of 3 parts^3^. Initially, the subjects were asked to name as many animals as possible in a minute. The next part was for the subjects to list the names of as many household items as possible in a minute. Finally, the subjects were asked to alternate the names of animals and household items as many times as possible in a minute. There will be no points given for items more than once, and there will be points given for the number listed. BNT involves displaying the subjects with 30 pictures and asking them to name each one^4^. One point was given for each picture correctly answered, and the total number of correct answers was recorded. The lower the scores of the two scales, the severer the language dysfunction.

### Attention

Attention was evaluated by the Symbol Digit Modalities Test (SDMT), the Trail Making Test (TMT)-A, as well as the Stroop Color-Word Test (SCWT)-A and SCWT-B.

The beginning of the SDMT form are 9 unique geometric shapes paired with numbers ranging from 1 to 9. Below the key are rows of boxes with geometric shapes in the top boxes with rows of empty boxes. The subjects were instructed to fill each empty box with the number that matches the shape using the key at the top of the page. To start, the subjects completed 10 practice items. Following the practice trial, the subjects were then instructed to fill as many boxes as possible in 90 seconds, matching numbers to the corresponding shape^5^.

TMT consists of two parts, TMT-A and TMT-B^6^. The TMT-A required the subjects to draw a line between 25 consecutive numbers as quickly as possible without lifting their pencil. In TMT-B, except for the number 1, all the other 24 numbers appeared twice, which were square and circle shapes，respectively. The subjects were asked to connect 25 consecutive numbers in sequence as quickly as possible without lifting their pencil, interconnecting the two shapes while connecting the numbers.

SCWT consists of three parts, including SCWT-A, SCWT-B and SCWT-C^7^. In SCWT-A, there are 50 words in the SCWT-A, including red, yellow, blue and green. The subjects were asked to read each word correctly and quickly. In SCWT-B, there are 50 circles with four colors of red, yellow, blue and green. The subjects were asked to read each color accurately and rapidly from left to right. In SCWT-C, there are 50 words of different colors that did not correspond to color, and subjects were asked to read the color of the words instead of the words itself.

The longer it took to complete the test and the lower the score of the test, the worse the attention of the individual patient.

### Visuospatial ability

In RFT, subject was asked to duplicate a complex figure within 10 minutes, with scores named RFT-imitation, which was used to evaluate visuospatial ability^2^. A low score of RFT-imitation suggested compromised visuospatial ability.

### Executive function

Executive function was evaluated by the SCWT-C and the TMT-B. The low scores of the tests indicated impaired executive function.

# References

1. Guo Q, Zhao Q, Chen M, Ding D, Hong Z. A comparison study of mild cognitive impairment with 3 memory tests among Chinese individuals. *Alzheimer disease and associated disorders*. 2009;23(3):253-9.

2. Shin MS, Park SY, Park SR, Seol SH, Kwon JS. Clinical and empirical applications of the Rey-Osterrieth Complex Figure Test. *Nature protocols*. 2006;1(2):892-9.

3. Mok EH, Lam LC, Chiu HF. Category verbal fluency test performance in chinese elderly with Alzheimer's disease. *Dementia and geriatric cognitive disorders*. 2004;18(2):120-4.

4. Katsumata Y, Mathews M, Abner EL, et al. Assessing the discriminant ability, reliability, and comparability of multiple short forms of the Boston Naming Test in an Alzheimer's disease center cohort. *Dementia and geriatric cognitive disorders*. 2015;39(3-4):215-27.

5. Fellows RP, Schmitter-Edgecombe M. Symbol Digit Modalities Test: Regression-Based Normative Data and Clinical Utility. *Archives of clinical neuropsychology : the official journal of the National Academy of Neuropsychologists*. 2019;35(1):105-115.

6. Wei M, Shi J, Li T, et al. Diagnostic Accuracy of the Chinese Version of the Trail-Making Test for Screening Cognitive Impairment. *Journal of the American Geriatrics Society*. 2018;66(1):92-99.

7. Bondi MW, Serody AB, Chan AS, et al. Cognitive and neuropathologic correlates of Stroop Color-Word Test performance in Alzheimer's disease. *Neuropsychology*. 2002;16(3):335-43.

# Supplementary Tables

**Supplementary Table S1 Association between Aβ_1-42_ level in CSF and cognitive function in AD patients**

|  | **Unadjusted** | | **Adjusted** | |
| --- | --- | --- | --- | --- |
|  | β (95%CI) | *P* | β (95%CI) | *P* |
| MMSE (points) | 3.51(1.51, 5.52) | **<0.001**** | 3.62 (1.56, 5.69) | **<0.001**** |
| MoCA (points) | 4.03 (2.21, 5.86) | **<0.001**** | 3.68 (1.84, 5.53) | **<0.001**** |
| AVLT N1-3 (points) | 2.25 (0.63,3.88) | **0.007**** | 2.31 (0.53, 4.10) | **0.012*** |
| AVLT N4 (points ) | 1.05 (0.29, 1.81) | **0.007**** | 1.09 (0.29, 1.90) | **0.008**** |
| AVLT N5 (points) | 0.60 (-0.09, 1.28) | 0.086 | 1.00 (0.26, 1.75) | **0.009**** |
| RFT delayed recall (points) | 3.39 (0.79, 5.99) | **0.011*** | 3.51 (0.75, 6.27) | **0.014*** |
| VFT (points) | 5.44 (1.39, 9.48) | **0.009**** | 6.11 (1.57, 10.64) | **0.009**** |
| SDMT (points) | 7.97 (1.58, 14.36) | **0.015*** | 10.05 (2.20, 17.90) | **0.013*** |
| RFT imitation (points) | 4.85 (0.80, 8.91) | **0.019*** | 4.17 (-0.71, 9.05) | 0.093 |

Age, gender, disease duration and education level were adjusted. Abbreviation: Aβ, β amyloid protein; AD, Alzheimer’s disease; MMSE, Mini-Mental State Examination; MoCA, Montreal Cognitive Assessment; AVLT, Auditory Verbal Learning Test; RFT, Rey-Osterrieth Complex Figure Test; VFT, Verbal Fluency Test; SDMT, Symbol Digit Modalities Test; RFT, Rey-Osterrieth Complex Figure Test. **P* < 0.05*, **P* < 0.01.

**Supplementary Table S2 Association between NO level in CSF and cognitive function in AD patients**

|  | **Unadjusted** | | **Adjusted** | |
| --- | --- | --- | --- | --- |
|  | β (95%CI) | *P* | β (95%CI) | *P* |
| TMT-A-time (seconds) | 2.44 (0.18, 4.70) | **0.034*** | 3.24 (0.00, 6.47) | 0.050 |
| TMT-B-time (seconds) | 5.11 (2.53, 7.70) | **<0.001**** | 5.24 (1.11, 9.37) | **0.013*** |

Age, gender, disease duration and education level were adjusted. Abbreviation: NO, nitric oxide; AD, Alzheimer’s disease; TMT, Trail Making Test. **P* < 0.05*, **P* < 0.01.

**Supplementary Table S3 Association between IL-1β level in CSF and cognitive function in AD patients**

|  | **Unadjusted** | | **Adjusted** | |
| --- | --- | --- | --- | --- |
|  | β (95%CI) | *P* | β (95%CI) | *P* |
| VFT (points) | -0.95(-1.92, 0.02) | 0.055 | -0.81(-2.27, 0.66) | 0.277 |
| SCWT-A-time (seconds) | 4.17(0.64, 7.70) | **0.021*** | 8.10(2.51, 13.68) | **0.005**** |
| SCWT-B-time (seconds) | 2.00(-0.86, 4.87) | 0.17 | 3.64(-1.07, 8.36) | 0.128 |
| RFT imitation (points) | -0.99(-2.05, 0.08) | 0.069 | -0.27 (-1.78, 1.24) | 0.725 |
| SCWT-C-time (seconds) | 7.14(2.92, 11.35) | **0.001**** | 6.61(0.27, 12.94) | **0.041*** |

Age, gender, disease duration and education level were adjusted. Abbreviation: IL-1β, interleukin-1β; AD, Alzheimer’s disease; VFT, Verbal Fluency Test; SCWT, The Stroop Color and Word Test; RFT, Rey-Osterrieth Complex Figure Test. **P* < 0.05*, **P* < 0.01.

**Supplementary Table S4 Association between sTREM-2 level in CSF and cognitive function in AD patients**

|  | **Unadjusted** | | **Adjusted** | |
| --- | --- | --- | --- | --- |
|  | β (95%CI) | *P* | β (95%CI) | *P* |
| VFT (points) | -0.01 (-0.02, 0.00) | 0.050 | -0.00 (-0.01, 0.00) | 0.358 |

Age, gender, disease duration and education level were adjusted. Abbreviation: sTREM-2, triggering receptor expressed on myeloid cells-2; AD, Alzheimer’s disease; VFT, Verbal Fluency Test.

**Supplementary Table S5 Association between YKL-40 level in CSF and cognitive function in AD patients**

|  | **Unadjusted** | | **Adjusted** | |
| --- | --- | --- | --- | --- |
|  | β (95% CI) | *P* | β (95% CI) | *P* |
| BNT （points) | -9.30E-5 (-1.84E-4, -1.00E-6) | **0.048*** | -1.30E-4 (-2.26E-4, 3.30E-5) | **0.011*** |
| TMT-B-time (seconds) | -5.61E-4 (-1.19E-3, 6.60E-5) | 0.078 | -7.18E-4 (1.59E-3, 1.50E-4) | 0.101 |

Age, gender, disease duration and education level were adjusted. Abbreviation: YKL-40, Tyr-Lys-Leu-40; AD, Alzheimer’s disease; BNT, Boston Naming Test; TMT, Trail Making Test. **P* < 0.05*.*
